# Influence of Subclinical Ketosis in Dairy Cows on Ingestive-Related Behaviours Registered with a Real-Time System

**DOI:** 10.3390/ani10122288

**Published:** 2020-12-03

**Authors:** Ramūnas Antanaitis, Vida Juozaitienė, Mindaugas Televičius, Dovilė Malašauskienė, Mingaudas Urbutis, Walter Baumgartner

**Affiliations:** 1 Large Animal Clinic, Veterinary Academy, Lithuanian University of Health Sciences, Tilžės str 18, LT-47181 Kaunas, Lithuania; mindaugas.televicius@lsmuni.lt (M.T.); dovile.malasauskiene@lsmuni.lt (D.M.); mingaudas.urbutis@lsmuni.lt (M.U.); 2 Department of Animal Breeding, Veterinary Academy, Lithuanian University of Health Sciences, Tilžės str 18, LT-47181 Kaunas, Lithuania; vida.juozaitiene@lsmuni.lt; 3 University Clinic for Ruminants, University of Veterinary Medicine, Veterinaerplatz 1, A-1210 Vienna, Austria; walter.baumgartner@vetmeduni.ac.at

**Keywords:** rumination, dairy cows, subclinical ketosis, RumiWatch System

## Abstract

**Simple Summary:**

Earlier disease detection may benefit cows by improving responses to treatment. We expected that changes in rumination before evident clinical signs of subclinical ketosis would result in the earlier identification of disease. Cows with subclinical ketosis showed lesser average values for the following parameters: rumination time and rumination chews (1.48 and 1.68 times respectively; *p* < 0.001), drinking time (1.50 times; *p* < 0.001), chews per minute, bolus and chews per bolus (1.12, 1.45 and 1.51 times; *p* < 0.001). From the 15th day before the diagnosis of ketosis, rumination time in the healthy group was greater than that in subclinical ketosis cows from −0.96% (−17 day) to 187.79% (0 days, *p* < 0.001). Eating time at the beginning of the experiment in healthy cows was 48.92% and at the end of the period was −91.97% lesser compared to subclinical ketosis (*p* < 0.001).

**Abstract:**

According to the literature, rumination time can be used as biomarker in the diagnosis of subclinical ketosis (SCK). We hypothesized that SCK in cows influences ingestive-related behaviours registered with the real-time system. The aim of the current study was to determine the influence of SCK on dairy cows’ ingestive-related behaviours registered with a real-time system. Twenty Lithuanian Black and White breed dairy cows were selected based on the following criteria: First day after calving, having two or more lactations (on average 3.0 ± 0.13 lactations), and being clinically healthy. The experiment lasted 18 days. Cows were tested 24 h a day for 17.5 days. On the day of diagnosis (day 0), data were recorded for 12 h. During the experimental period, one cow was studied for a total of 420 h. For the registration of rumination behaviour, the RumiWatch system (RWS) was used. It was found that cows with SCK showed lesser average values for the following parameters: rumination time and rumination chews (1.48 and 1.68 times respectively; *p* < 0.001), drinking time (1.50 times; *p* < 0.001), chews per minute, bolus and chews per bolus (1.12, 1.45 and 1.51 times; *p* < 0.001). From the 15th day before the diagnosis of SCK, rumination time in health cows was greater than that in SCK cows from −0.96% (−17 day) to 187.79% (0 days, < 0.001). We estimated the greater average value of drinking time in healthy cows compared with SCK cows from 34.22% on day −17 to −121.67% on day 0 (*p* < 0.001). Decrease in rumination time was associated with a significant increase in the probability of risk of SCK. Further studies are needed with a larger number of cows with SCK.

## 1. Introduction

The dairy industry has developed and implemented multiple devices for the automated monitoring of behavioural and physiological parameters [1]. A lot of important information about the productivity, welfare and especially health of dairy cows can be acquired by monitoring ingestive-related behaviours such as feeding and ruminating [2]. For example, changes in the duration of feeding and rumination can indicate a decline in cow comfort and welfare [3]. According to Kovács et al. [4], rumination is considered to be one of the main indicators of animal welfare and is impactful to the physiological health of ruminants. After investigations by Urton et al. [5], rumination time in both pre- and post-partum periods, was lesser for cows diagnosed with clinical and subclinical metritis compared to healthy cows. Therefore, to keep with animal welfare and health standards, automated monitoring of rumination is of particular importance [6]. With the extent of modern animal farming rapidly increasing and advances made in the research of precision animal husbandry, the monitoring of rumination in real-time has been made possible [6] with the emergence of new methods and sophisticated equipment [7]. The core device of the monitoring equipment has a built-in pressure sensor, which can identify rumination by tracking pressure changes and masticatory intervals of the temporal fossa or noseband during chewing. The effectiveness of automated rumination monitoring can be seen in the results where correlation coefficients between the rumination duration obtained by automatic monitoring and direct observation were 0.99 and 0.93, respectively [8,9]. Werner et al. [10] assessed the noseband pressure equipment effect on rumination monitoring in grazing cows. The results showed that the Concordance Correlation Coefficient (CCC) of rumination duration per hour was 0.93, and the monitoring accuracy of rumination times per hour CCC was 0.86. Andriamandroso et al. [11] mainly stated that rumination and other behaviours were identified by peak mode and peak interval of time domain rumination noseband pressure signals. Hirayama et al. [12] distinguished between feeding, rumination, and other behaviours with the use of jaw movement sensors. Uncomplicated decision-tree algorithms manage to classify ruminating and feeding behaviours [2]. It would enable online measurements of the ingestive-related cow behaviours [2]. The RumiWatch (ITIN + HOCH GmbH, Fütterungstechnik, Liestal, Switzerland) noseband sensor was developed and validated as a device for the monitoring of rumination and eating activities in stable-fed dairy cows [13]. The RumiWatch noseband sensor per se can be regarded as an advanced technique to monitor jaw movements [14]. Data, generated by the sensor, are sufficient enough to identify health disorders in cows, but can also be used with well-known monitoring protocols [15,16]. Furthermore, thorough monitoring of physiological and behavioural parameters may allow for the detection of minute changes before the appearance of clear clinical signs. Earlier diagnoses could benefit cows by preventing disease progression and improving their response to treatment [17]. Preliminary findings of Benaissa et al. [2] illustrate the potential of the widely used collar-mounted accelerometer to classify ruminating and feeding behaviours with accuracy measures closely comparable to the RumiWatch noseband sensor.

The early post-partum period of lactating dairy cows is substantially affected by health disorders, which negatively influences their performance, welfare, and health [18]. McArt et al. [19] reported an average cumulative subclinical ketosis incidence of 43% among cows tested thrice weekly from 3 to 16 days in milk (DIM), with the peak incidence at five DIM. The current challenge for producers is identifying subclinical ketosis (SCK) and abomasal displacement at an early stage [20,21]. Additional tools to confirm the effect of subclinical disorders or underlying predisposing factors for clinical diseases may influence decision-making [19]. Future research is set on establishing criteria for the differentiation and treatment of particular health disorders according to the information provided by the automatic health monitoring system (AHMS) [17]. Signs of this, that rumination time can be used as biomarker in the diagnosis of subclinical ketosis, are clear. Future studies with a larger number of animals, however, are needed [22].

We hypothesized that SKC in cows influences ingestive-related behaviours registered with the real-time system. Additionally, we expected that changes in rumination before evident clinical signs of SCK could be used for early identification of the disease. The aim of the current study was to determine the impact of subclinical ketosis in dairy cows on ingestive-related behaviours registered with a real-time system.

## 2. Materials and Methods 

### 2.1. Location, Animals, and Experimental Design

The study was carried out in southern Lithuania (46°45′59.0″ N, 7°6′17.2″ E) from 1 June 2020 to 8 August 2020. Prior to selection of the cows, all possible cow candidates underwent a veterinary check-up. Twenty from 120 Lithuanian Black and White breed dairy cows were selected based on the following criteria: having two or more lactations (on average 3.0 ± 0.13 lactations) and being clinically healthy. The experiment lasted for 18 days. Cows were tested 24 h a day for 17.5 days. On the day of diagnosis (day 0), data were recorded for 12 h. During the experimental period, one cow was studied for a total of 420 h. The study measurements began 30 days post-partum. Ten of the cows were never diagnosed with SCK.

The cows resided in a free stall barn and were fed a total mixed ration (TMR) routinely throughout the year, balanced according to their physiological needs. Feeding of cows took place every day at 06:00 and 18:00 with a typical total mixed ration for high-producing, multiparous cows that consisted primarily of 50% grain concentrate mash, 6% alfalfa hay, 10% grass silage, sugar beet pulp silage, 30% corn silage, 4% grass hay wheat straw, and compound feed. Diets were formulated accordingly to meet or exceed the requirements of a 550 kg Holstein cow producing 35 kg/d. Chemical composition of ration: dry matter (DM) (%) 48.8; neutral detergent fibre (% of DM) 28.2; acid detergent fibre (% of DM) 19.8; nonfibre carbohydrates (% of DM) 38.7; crude protein (% of DM) 15.8; net energy for lactation (Mcal/kg) 1.6. The cows were milked twice per day through a parlour system at 05:00 and 17:00. The average body weight of the cows was 550 kg +/−45 kg. Cows were housed in ventilated free-stall barns. The average energy corrected milk yield (4.2% fat, 3.5% protein) in 2019 was 9.500 kg per cow per lactation.

The research was carried out in accordance with the provisions of the Law on Animal Welfare and Protection of the Republic of Lithuania (Official Gazette Valstybės žinios, 1997, No. 108-2728; 2012, No. 122-6126). 22 September Directive 2010/63/EU of the European Parliament and of the Council on the protection of animals used for scientific purposes (OJ 2010 L 276, p. 33) and having regard to the European Convention for the protection of vertebrate animals used for experimental and other scientific purposes (Official Journal 2007, No 49) -1883, No. 49-1884). The study approval number was PK016965.

#### 2.1.1. Subclinical Ketosis Group (SCK)

Ten from 120 cows were classified as being SCK when at least one beta-hydroxybutyrate (BHB) reading during the 30-day post-partum period was at ≥1.2 mmol/L. Milk fat/ protein ratio (F/P) for that group of cows was registered as >1.2. They were without any clinical sign of other diseases after calving (metritis, lameness, mastitis, displaced abomasum, indigestion with an average rectal temperature of +38.8 °C, rumen motility five–six times per three minutes).

#### 2.1.2. Healthy Group (HG)

Ten from 120 cows that showed no clinical signs of disease after calving and all of their BHB readings in the 30-day period post calving were at <1.2 mmol/L, were classified in this group. The average milk F/P for this group of cows was at 1.2.

The two groups of animals (SCK and HG) were allocated together.

### 2.2. Measurements

The RumiWatch System (RWS) consists of a noseband halter that has a liquid-filled pressure tube and a built-in pressure detector. The pressure in the tube is changed with each chewing stroke, when the curvature of the pressure tube is mechanically deformed. The pressure signal generated by the pressure sensor is transmitted to the data logger, which is safely stored in a hard plastic box on the halter. An acceleration sensor that detects triaxial head movements, together with a secure digital cardholder for data storage with a Universal Serial Bus interface, are incorporated as well. Binary files containing the pressure and acceleration data are saved at a 10 Hz frequency. A wireless data transmitter establishes a connection between the halter and the RumiWatch Manager software to acquire live data. RWS software uses generic algorithms to process the customised classification of behavioural characteristics from 10 Hz pressure data in different preferred time summaries, e.g., 1 min and 1 h, whereby each time summary is classified by a specific algorithm. The consolidation of 10 Hz pressure data in 1-min summaries only facilitates a binary output of the behavioural characteristics of rumination, eating, drinking and other activity, executed within the respective minute or not, therefore further behavioural parameters are convertible from raw pressure data in 1-h summaries (Table 1). The classifications performed by the aforementioned algorithms are based on the identification of specific pressure peak profiles, produced by the movements of the jaw, which are differentiated between the behavioural characteristics [13]. In particular, the behaviour classification takes into account the frequency of pressure peaks and not the height of pressure amplitudes. Furthermore, the algorithms calculate the duration of the respective pressure peak profiles and identify pauses, in which, e.g., a rumination bolus is swallowed. The binary classification result of the 1-min time summary only concentrates and displays the behavioural characteristic which was performed the longest time during that minute. 

### 2.3. Data Analysis and Statistics

The statistical analysis of RumiWatch data was performed with the SPSS 20.0 (SPSS Inc., Chicago, IL, USA) program package. Using descriptive statistics, normal distributions of variables were assessed using the Kolmogorov–Smirnov test. The results were produced as the mean ± standard error of the mean (M ± SE). Differences between SKC and HG in the mean values of normally distributed variables were analysed using the Student’s *t*-test. 

A linear regression equation was calculated to determine the statistical relationship between days (from day −17 to day 0) of the experiment (independent variable) and RumiWatch (dependent) variables.

The Pearson correlation coefficient was calculated to define the linear relationship between ruminant time and other RumiWatch variables.

For the Chi-squared (χ^2^) test and binary logistic regression analysis, rumination time data were divided into 6 classes: <10 min, 10–20 min, >20–30 min, >30–40 min, >40–50 min, and >50–60 min. The 95% Wald limit was used to calculate the odds ratio (OR) and the 95% confidence interval (CI) in the logistic regression analysis.

## 3. Results

On average, the diagnosis of subclinical ketosis was confirmed 10 days into the experiment.

### 3.1. Influence of Health Status of Cows on Ingestive-Related Behaviours

SCK cows showed lesser average values for the following parameters: Rumination time and rumination chews drinking time chews per minute, boluses, and chews per bolus). However, the arithmetic mean of HG cows was found to be lesser for the following indicators: Drinking gulp), eating time and eating chews (Table 2).

### 3.2. Changes in Ingestive-Related Behaviours Indicators During the Experiment by Group of Cows

**Figure 1:**
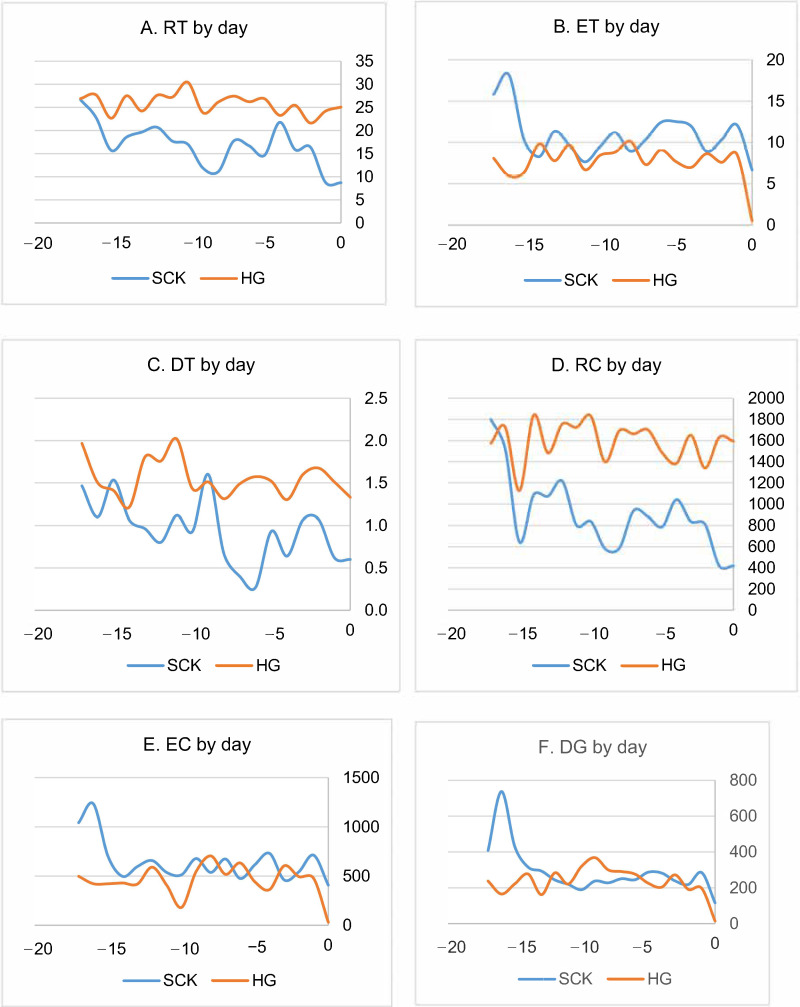

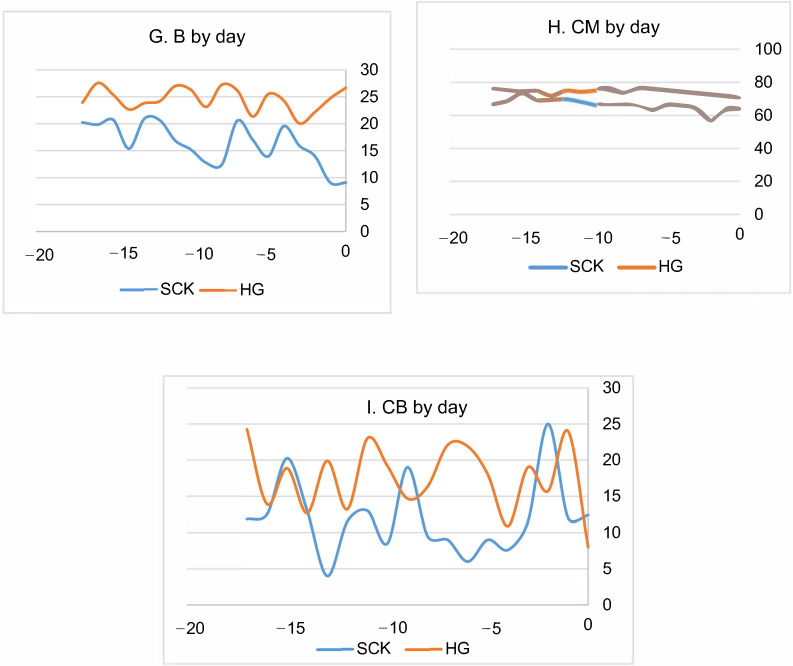
Results of RumiWatch noseband sensor readings. Values are the means of 10 SCK and HG dairy cows observed in summer 2020 in Lithuania. RT—Rumination time (time in minutes spent for rumination chews); ET—Eating time (time in minutes spent for eating chews); DT—Drinking time (time in minutes spent for drinking); RC—Rumination chews (chews during rumination for mechanical breakdown of the regurgitated materials); EC—Eating chews (number of prehension bites); DG— Drinking gulp (total number of drinking gulps while drinking); B—Bolus (a regurgitated mass of cud); CM—Chews per minute (chews for one minute); CB—Chews per bolus (chews performed during rumination); SCK—Subclinical ketosis group; HG—healthy group.

From the 15th day before the diagnosis of ketosis, rumination time in HG was greater than that in SCK cows from −0.96% (−17 day) to 187.79% (0 days, *p* < 0.001). Eating time at the beginning of the experiment in HG was 48.92% and at the end of the period it was 91.97% less compared to SCK (*p* < 0.001). ET at the start of the experiment in HG was 14.32% and at day 0 was 73.06% less compared to SCK (*p* < 0.001). We estimated the greater average value of drinking time in HG from 34.22% on the day −17 to −121.67% on day 0 (*p* < 0.001). The difference in rumination chews between groups increased during the experiment from 12.52% (day −17) to 280.05–288.87 % (day−1–day 0) (*p* < 0.001). On day 0, indicators of eating chews and drinking gulp in HG were 92.40% and 87.50% lesser compared to SCK (*p* < 0.001), on the other hand bolus (194.12%, *p* < 0.001), chews per minute (9.76%, *p* < 0.001) were statistically significantly greater. Chews per bolus (35.48%, *p* < 0.001) on day 0 were lesser in HG (Figure 1).

A description of the change in the observed indicators during experimental days according to the linear regression equation is given in the Table 3. The analysis showed that the change of eight indicators (RT, ET, DT, EC, RC, DG, B and CM) during the experiment for SCK cows can be described by a linear regression equation with a statistically reliable regression coefficient and R^2^ from 0.2903 (for EC changes) to 0.4970 (for CM), while in HG cows a statistically reliable coefficient of regression was found for only one indicator—CM (*p* = 0.046) but R^2^ (0.2263) did not show the possibility of applying linear regression. 

### 3.3. Relationship of Rumination Time with Other Ingestive-Related Behaviour Indicators and Health Status of Cows

**Figure 2:**
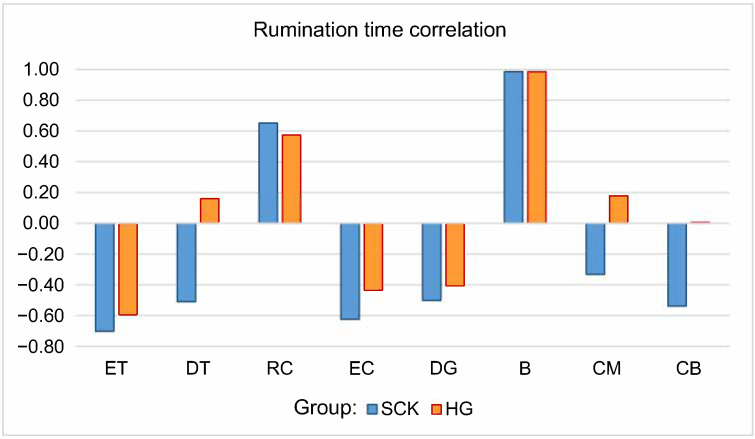
Correlation coefficients of rumination time with other noseband sensor readings of dairy cows that were eventually diagnosed with subclinical ketosis and cows that remained healthy in 2020 in Lithuania by groups of cows. RT—Rumination time), ET—Eating time, DT—Drinking time, RC—Rumination chews, EC—Eating chews, DG—Drinking gulp, B—Bolus, CM—Chews per minute, CB—Chews per bolus; SCK—Subclinical ketosis group, HG—healthy group.

The rumination time in both groups of cows (Figure 2) was statistically significantly negatively related to ET, EC, and DG, and positively related to RC and B (*p* < 0.05). The rumination time showed a dependence with DT and CM indicators in the opposite direction for groups of cows—a positive correlation in HG cows and a negative relationship in the SCK group (*p* < 0.05).

**Figure 3:**
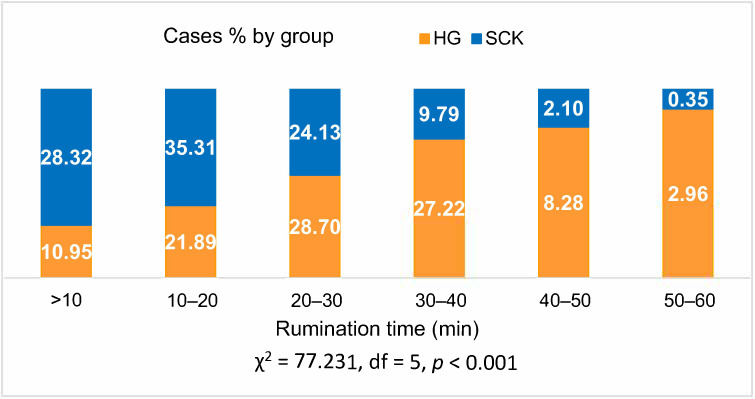
Relationship of rumination time (min) registered by RumiWatch noseband sensor readings between dairy cows of different health status in 2020 in Lithuania. SCK—Subclinical ketosis group, HG— healthy group.

The rumination time of healthy cows of <20 min was found in 32.84% of cases, and in SCK cows in 63.63% of cases; rumination time ≥40 min was found in 11.24% of healthy cows and 2.45% of SCK cows (*p* < 0.001; Figure 3). 

A binary regression showed that a decrease in rumination time of cows was associated with a significant increase in the probability of risk of the SCK (OR = 1.897; 95% CI = 1.897–2.209, *p* < 0.001).

## 4. Discussion

Ketosis has a major influence on the occurrence of abomasal displacement (AD) in lactating dairy cows [21,23]. Automated health-monitoring systems in dairy farms that register activity and rumination are promising tools for the identification of cows with digestive and metabolic disorders [17]. Monitoring the time of eating and rumination is becoming a trend in modern dairy farming [24]. A comprehensive health-monitoring program is required to quickly detect cows with health disorders in the early post-partum period [17]. Detecting a health disorder as early as possible and before the manifestation of clear clinical signs may be beneficial to the cows if the overall treatment response is improved and the long-term negative consequences of disease on overall cow performance and health are reduced [23]. Automatic monitoring of rumination and feeding behaviour in transition dairy cows shows promise for the detection of health problems. Moreover, a combination of both behaviours would likely result in increased detection rates. Monitoring individual rumination behaviour with the system used in our study is easier than monitoring individual feed intake and might be more practical and therefore more likely to be implemented on commercial farms. Rumination time alone, or when combined with other variables, has been investigated as a means of detecting parturition and illness in dairy cows on commercial farms [15,25]. Studies have also examined whether rumination activity is consistently lesser in sick compared with healthy cows [26].

In this study, we characterised the changes in rumination behaviour before and during SCK. We found that cows with SCK had a lower rumination time and drinking time, fewer rumination chews, chews per minute, boluses (swallowed while eating or regurgitated during rumination) and chews per bolus. Previous studies have stated that rumination time was closely related to clinical and subclinical health disorders [27,28,29]. Dramatic reductions in rumination [28] five to six days before the diagnosis of ketosis in dairy cows was also reported. Considering the earlier identification of cows with health disorders, it presents both opportunities and challenges [17]. Cows diagnosed with SCK have been observed to have lesser rumination time than healthy cows in the first week after calving [27,29]. Rumination behaviour may be a promising indicator of metabolic conditions [27], particularly during the post-partum period because it is likely affected by changes in feeding behaviour [30]. According to our results, from the 15th day before the diagnosis of SCK, rumination time and drinking time in HG was greater than that in SCK. The values of these indicators in the SCK group of cows in the 17-day period before the diagnosis of the disease changed according to the linear regression equation (y = −0.5824x + 22.33; R^2^ = 0.4311 for RT, and y = −0.0378x + 1.2949; R^2^ = 0.3001 for DT), while in the HG group the change of these parameters did not show a linear trend.

Kaufman et al. [20] reported that rumination monitoring across the transition period may contribute to the identification of multiparous cows either at risk of developing SCK or those that suffer from SCK in combination with other health problems. To use rumination data to help identify multiparous cows at risk for developing subclinical ketosis after calving, it is important to begin monitoring rumination during the dry period to establish a baseline for each cow. In our study, we expected that changes in rumination before evident clinical signs of SCK would be used for earlier identification of the disease.

In the present study, we found evidence that a decrease in rumination time was associated with a significant increase in the probability of risk of the SCK, although Toni et al. [29] did not comment on an association between SCK and precalving or postcalving daily rumination time. Kaufman et al. [20] reported that lower rumination times were observed in multiparous SCK cows during the pre- and post-partum period compared with healthy cows. According to Soriani et al. [27] cows with the shorter rumination time showed a greater incidence of clinical disease (including mastitis, lameness, ketosis, and AD). Low dry matter intake (DMI) and reduced feeding time are considered important risk factors for SCK. Studies by Antanaitis et al. [31] and Goldhawk et al. [32] observed a 3 kg reduction from 19 kg in daily DMI during the week before being diagnosed with ketosis. Shorter rumination times may be indicative of low DMI in the pre-partum period [33]; however, many cow-level and management-related factors vary between farms and have a great effect on rumination time [20]. Rumination behaviour could be a promising indicator to track metabolic conditions associated with a decrease in DMI [27], such as subclinical ketosis (SCK). The time dairy cattle spend eating is highly variable when combined across experimental conditions. White et al. [34] reported a mean eating time of 284 min/d ranging from 141 to 507 min/d. Part of the variation may be due to the slightly different criteria used among studies to define eating time, but eating time is also highly affected by feed management, DMI, physical and chemical composition of the diet, and inherent variability among animals [34]. There are signs of a compensatory relationship between ruminating and eating time. Dado and Allen [35] mentioned that for dairy cows with unrestricted feed access, the correlation coefficient between eating time and ruminating time was −0.62, indicating that cows which are spending less time eating have a tendency to ruminate longer. Predicting the time that cows spend chewing or ruminating can be a valuable management tool in terms of optimising cow health, but the accuracy can be low because of the many interacting factors [26]. The difference in rumination time between sick and healthy cows appears to depend on the disease, the period relative to calving, and the season [36]. King et al. [37] reported that rumination time declined by 45 min/d from eight days before a diagnosis of DA, by 25 min/d from six days before subclinical ketosis and by 50 min/d from five days before pneumonia. Kaufman et al. [20] found that primiparous cows showed no change in rumination time in relation to the incidence of ketosis, possibly due to the limited numbers of primiparous cows with ketosis. Schirmann et al. [38] described that compared with healthy cows, those with subclinical ketosis and ketosis plus metritis had lesser pre-partum DMI and continued to eat less for two to three weeks post-partum, but rumination was decreased only in cows with ketosis precalving. Paudyal et al. [36] observed that for cows with ketosis, rumination time was reduced both pre- and post-partum in both seasons [36]. Paudyal et al. [36] expected to see an increase in drinking time post-partum, reaching a maximum minutes per day in early-lactation, however our observations show a clear reduction (eight min/d during early-lactation). However, drinking time must be considered critically—even if it is shorter, more water might be ingested if the gulps are larger or more frequent. Moreover, many factors can have an effect on water consumption: changes in ambient temperatures, increased loss of water due to increased milk production, amount of feed ingested, consumption of sodium and potassium, dry matter of the diets, as well as physiological factors and diseases [39].

## 5. Conclusions

Cows with subclinical ketosis had lesser rumination time and rumination chews, drinking time, chews per minute, boluses, and chews per bolus. Seventeen days before diagnosis of SCK, rumination time and drinking time in HG was greater than that in SCK. Decrease in rumination time was associated with a significant increase in the probability of risk of the SCK. According to the results of our study, we can conclude that changes in rumination before evident clinical signs of SCK could be used for early identification of the disease. Further studies are needed with a larger number of cows with SCK.

## Figures and Tables

**Table 1:** Behavioural characteristics of cattle afflicted by subclinical ketosis as measured by the RumiWatch noseband sensor (ITIN + HOCH GmbH, Fütterungstechnik, Liestal, Switzerland).

Characteristic	Definition
**Rumination time (RT)**	Time spent for rumination chews including chewing interruptions up to 5 s.
**Eating time (ET)**	Time spent for eating chews, including interruptions between eating chews up to 5 s.
**Drinking time (DT)**	Time spent for drinking, including interruptions between drinking gulps up to 5 s.
**Rumination chews (RC)**	Chews during rumination for mechanical breakdown of the regurgitated materials into finer particles using the molars.
**Eating chews (EC)**	Total number of prehension bites and mastication chews while eating.
**Drinking gulps (DG)**	Total number of drinking gulps while drinking.
**Bolus (B)**	Number of boluses per rumination
**Chews per minute (CM)**	Chews for one minute.
**Chews per bolus (CB)**	Chews performed during rumination between the regurgitation and swallowing of 1 bolus.

**Table 2:** Results of RumiWatch noseband sensor readings of dairy cows that were eventually diagnosed with subclinical ketosis and cows that remained healthy in 2020 at Lithuania. Means (M) and standard errors (SE) by groups of cows.

Characteristics *	SCK		HG		*p*
	M	SE	M	SE	
**RT**	17.43	0.613	25.77	0.673	<0.001
**ET**	11.08	0.486	7.97	0.439	<0.001
**DT**	1.03	0.078	1.55	0.07	<0.001
**RC**	943.67	40.569	1586.41	49.667	<0.001
**EC**	656.63	34.763	471.74	29.747	<0.001
**DG**	300.64	17.754	243.91	16.576	0.021
**B**	16.84	0.719	24.44	0.765	<0.001
**CM**	66.56	0.614	74.33	0.303	<0.001
**CB**	11.84	1.102	17.88	0.945	<0.001

* RT—Rumination time (time in minutes spent for rumination chews); ET—Eating time (time in minutes spent for eating chews); DT—Drinking time (time in minutes spent for drinking); RC—Rumination chews (chews during rumination for mechanical breakdown of the regurgitated materials); EC—Eating chews (number of prehension bites); DG—Drinking gulp (total number of drinking gulps while drinking); B—number of boluses per rumination) CM—Chews per minute (chews for one minute); CB—Chews per bolus (chews performed during rumination); SCK—Subclinical ketosis group, HG—healthy group.

**Table 3:** Evaluation of changes in RumiWatch noseband sensor readings observed in summer 2020 in Lithuania by day of the experiment (independent indicator) using linear regression.

Dependent Indicator	SCK			HG		
	y	R^2^	*p*	y	R^2^	*p*
**RT**	−0.5824x + 22.33	0.4311	0.003	−0.1499x + 27.231	0.1323	0.138
**ET**	−0.1958x + 12.756	0.1399	0.126	−0.1005x + 8.632	0.0631	0.315
**DT**	0.0378x + 1.2949	0.3001	0.019	0.0119x + 1.6641	0.0805	0.254
**RC**	−44.567x + 1328.3	0.4478	0.020	−3.6868x + 1623.8	0.011	0.679
**EC**	−20.732x + 841.79	0.2903	0.033	−3.6054x + 488.51	0.015	0.699
**DG**	−15.327x + 435.99	0.379	0.007	−3.7911x + 270.9	0.0674	0.298
**B**	−0.4902x + 20.991	0.4434	0.003	−0.0798x + 25.316	0.0401	0.425
**CM**	−0.4459x + 70.64	0.4970	0.001	−0.1295x + 75.458	0.2263	0.046
**CB**	0.0199x + 11.788	0.0004	0.935	−0.143x + 18.889	0.0266	0.518

RT—Rumination time, ET—Eating time, DT—Drinking time, RC—Rumination chews, EC—Eating chews, DG—Drinking gulp, B—Bolus, CM—Chews per minute, CB—Chews per bolus; SCK—Subclinical ketosis group, HG—healthy group.
